# Perceived Barriers and Facilitators of Using a Web-Based Interactive Decision Aid for Colorectal Cancer Screening in Community Practice Settings: Findings From Focus Groups With Primary Care Clinicians and Medical Office Staff

**DOI:** 10.2196/jmir.2914

**Published:** 2013-12-18

**Authors:** Masahito Jimbo, Cameron Garth Shultz, Donald Eugene Nease, Michael Derwin Fetters, Debra Power, Mack Thomas Ruffin IV

**Affiliations:** ^1^University of MichiganDepartments of Family Medicine and UrologyAnn Arbor, MIUnited States; ^2^University of MichiganDepartment of Family MedicineAnn Arbor, MIUnited States; ^3^University of ColoradoDepartment of Family MedicineAurora, COUnited States; ^4^Power Marketing ResearchAnn Arbor, MIUnited States

**Keywords:** colon cancer, colonoscopy, cancer screening, early detection of cancer, reminder systems, decision support techniques, focus groups, health information technology

## Abstract

**Background:**

Information is lacking about the capacity of those working in community practice settings to utilize health information technology for colorectal cancer screening.

**Objective:**

To address this gap we asked those working in community practice settings to share their perspectives about how the implementation of a Web-based patient-led decision aid might affect patient-clinician conversations about colorectal cancer screening and the day-to-day clinical workflow.

**Methods:**

Five focus groups in five community practice settings were conducted with 8 physicians, 1 physician assistant, and 18 clinic staff. Focus groups were organized using a semistructured discussion guide designed to identify factors that mediate and impede the use of a Web-based decision aid intended to clarify patient preferences for colorectal cancer screening and to trigger shared decision making during the clinical encounter.

**Results:**

All physicians, the physician assistant, and 8 of the 18 clinic staff were active participants in the focus groups. Clinician and staff participants from each setting reported a belief that the Web-based patient-led decision aid could be an informative and educational tool; in all but one setting participants reported a readiness to recommend the tool to patients. The exception related to clinicians from one clinic who described a preference for patients having fewer screening choices, noting that a colonoscopy was the preferred screening modality for patients in their clinic. Perceived barriers to utilizing the Web-based decision aid included patients’ lack of Internet access or low computer literacy, and potential impediments to the clinics’ daily workflow. Expanding patients’ use of an online decision aid that is both easy to access and understand and that is utilized by patients outside of the office visit was described as a potentially efficient means for soliciting patients’ screening preferences. Participants described that a system to link the online decision aid to a computerized reminder system could promote a better understanding of patients’ screening preferences, though some expressed concern that such a system could be difficult to keep up and running.

**Conclusions:**

Community practice clinicians and staff perceived the Web-based decision aid technology as promising but raised questions as to how the technology and resultant information would be integrated into their daily practice workflow. Additional research investigating how to best implement online decision aids should be conducted prior to the widespread adoption of such technology so as to maximize the benefits of the technology while minimizing workflow disruptions.

## Introduction

Colorectal cancer (CRC) screening is recommended for all average-risk adults aged 50 years and over [1]. Several CRC screening options are available for average-risk adults, including stool blood test and colonoscopy [1]. While up-to-date CRC screening rates of asymptomatic adults aged 50-75 years in the United States have increased from 54% in 2002 to 64.5% in 2010 [2], millions of eligible people remain unscreened by any method [3]. Given evidence that no single CRC screening test is superior at reducing CRC mortality [1], current recommendations advise CRC screening should be based on each patient’s preference and chosen using shared decision making [1,3-5]. Decision aids may help to facilitate shared decision making by reducing patient decisional conflict, improving patient knowledge, and stimulating patients to be more active in decision making without increasing anxiety [6,7]. Studies utilizing decision aids on CRC screening have shown variable results, with seven showing an increase [6,8-14], one showing a decrease [6,15], and five showing no difference in CRC screening [6,16-20].

**Figure 1:**
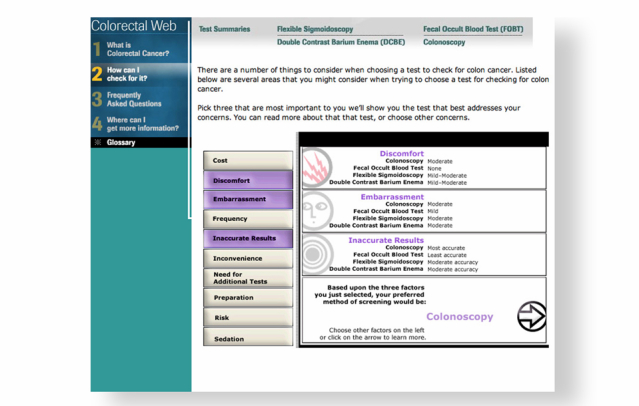
Colonoscopy selected as preferred screening method.

**Figure 2:**
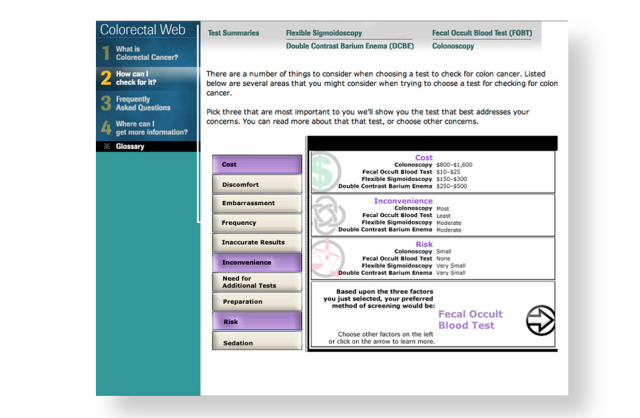
Fecal occult blood test selected as preferred screening method.

Colorectal Web was developed as a Web-based interactive decision aid to help adults aged 50 years and over make a choice among several medically appropriate CRC screening options [21,22]. In addition to helping patients understand their CRC risk by providing information on risk factors, a key feature of Colorectal Web is the interactive values clarification exercise where users identify their top three areas of concern from a menu of 10 concerns often cited about CRC screening: cost, discomfort, embarrassment, frequency, accuracy, convenience, additional testing, preparation, risk, and sedation. Figures 1 and 2 illustrate how Colorectal Web works. In the example illustrated by Figure 1, the user identified their three top concerns as frequency, accuracy, and need for additional tests. For this user, a colonoscopy would have been recommended because colonoscopies are recommended only every 10 years, have the best accuracy, and require no additional tests. In the example illustrated by Figure 2, the user identified their three top concerns to be cost, discomfort, and embarrassment. For this user, a stool blood test would have been recommended because stool blood tests are the most cost-effective screening tool, have the least discomfort, and are commonly perceived as the least embarrassing.

**Figure 3:**
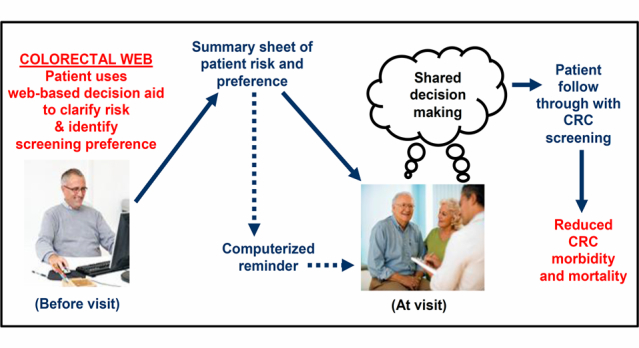
Linkage of colorectal Web to clinical encounter (solid lines show a sequence where the patients may print out their summary sheets to take to their clinicians; dotted lines show a sequence where the computerized reminder also generates the summary sheet for clinicians’ use).

As reported by Ruffin, Fetters, and Jimbo (2007), patients using Colorectal Web were more likely to follow through with CRC screening when compared to controls [10]. As summarized in Figure 3, we hypothesize that linking patients’ screening preferences directly to the clinical encounter would enable clinicians to more effectively engage patients in personalized conversation about CRC screening, which in turn would help improve patient-clinician communication, shared decision making, and patients’ CRC screening follow-through. We further posit that the combined use of the patient-led decision aid and clinician-directed computerized reminder system would help to streamline care in real world settings where efficiency and strict time management are essential. And finally, by improving patient-clinician communication, fostering shared decision making, and improving patient follow-through, we believe the effective use of the Web-based decision aid can help promote the early detection of CRC and improve patient outcomes.

Before these hypotheses can be tested, it is important to gain a better understanding of how those working in community practice settings perceive Web-based decision aids, a corresponding computerized reminder system, and the integration of these tools in the clinics’ daily operation. Of particular importance are the perceptions of clinicians and other staff regarding perceived facilitators and barriers to implementation, as well as how the tools might impact the clinical milieu. To this end, we conducted focus groups with both clinicians and nonclinical office staff to elucidate their perceptions, concerns, ideas, and opinions about the proposed use of Colorectal Web and the linking of patients’ identified screening preference to a computerized reminder system already piloted in each of the practice settings. We anticipate findings from this research will help inform policies that shape the development and implementation of Web-based decision aids, computerized reminder systems, and other health information technologies that promote patient activation, shared decision making, and a more patient centered patient-clinician encounter.

## Methods

### Design

This qualitative study utilized data collected from five focus groups involving both clinicians and key office staff from community practice settings. We employed focus groups because they are an efficient means to gather information while allowing participants to interact with each other as topics are explored. Allowing for give-and-take interactions between participants was critical for this research, as changes in clinical practice often affect the work of both clinicians and staff members alike. To adequately capture participants’ concerns, ideas, and opinions as to how the Web-based decision aid would affect the daily operations of the community-based practice, the perspectives of all key individuals who would likely be involved with implementation were invited to participate. Approval from the University of Michigan Institutional Review Board was obtained prior to the study’s execution.

### Setting

We conducted the focus groups in five community-based primary care settings across Michigan from August 2006 to May 2007.

### Sampling Frame

As the study’s purpose was to better understand the factors that mediate and impede the use of a Web-based CRC screening decision aid, it was decided to recruit from only those sites having previous experience using health information technologies. Such sites were also of interest because they likely typify early adopters of health information technology in community-oriented primary care. By focusing on these early adopters, we anticipated that reported barriers and facilitators would represent participants’ perspectives about the actual use of the Web-based tool rather than about the use of electronic health information technology per se. The reported barriers and facilitators would be based more on practical experience (ie, actual past use of health information technology) and less on assumptions (ie, guesses or presuppositions in the absence of previous use). To this end, we used an intentional sampling approach to recruit from 12 practice sites that previously had implemented a computerized reminder system as part of a National Cancer Institute-funded study examining the utility of a computerized reminder system to promote CRC screening [23]. Each site was affiliated with the Great Lakes Research Into Practice Network, a voluntary association of Michigan-based primary care practices that have expressed an interest in participating in health services research. Because clinicians and staff from these sites had previous experience using a computerized reminder system, they were uniquely positioned to provide an informed perspective on how the addition of the Web-based decision aid might affect the patient-clinician encounter and related processes of care.

Of the 12 candidate sites, one had previously indicated a desire to not participate in future research. Using the remaining 11 sites, a purposive recruitment strategy was employed to maximize variability in terms of practice size, location, and the site’s technological sophistication (eg, sites using and not using an electronic health record [EHR]). Based on their demographic profiles, seven sites were selected for recruitment. The study’s principal investigator (PI) subsequently contacted the clinical lead (either a lead physician or clinic manager) at each site by phone to discuss the aims of the study, the approach (ie, a focus group with clinic physicians and staff), and logistics (eg, the study’s time frame). Clinical leads from five sites expressed an interest in having their clinic participate.

### Discussion Guide Development

The semistructured discussion guide (available upon request) was developed to assess the four following areas: (1) current CRC screening approaches, (2) perceptions/opinions about Colorectal Web, (3) feasibility of implementing and linking the decision aid and computerized reminder system, and (4) anticipated influences on practice workflow and logistics. The discussion guide underwent several iterations until the study’s investigators reached 100% agreement.

### Recruitment

After the clinical leads at each of the five sites agreed to have their clinic participate, the focus group moderator scheduled focus groups for a time identified by the lead as convenient. All clinicians (physicians, and physician assistants), clinical support staff (registered nurses, licensed practical nurses, and medical assistants), and nonclinical support staff (clinic managers, receptionists, and billing clerks) were invited to participate in the focus group. Written informed consent was secured from each participant prior to the start of the focus group. While all clinicians and support staff from each site were invited to participate, some clinicians or staff by choice or circumstance may not have been present at the time of scheduled visit. Of those onsite at the time of the scheduled visit, all joined the focus group.

### Data Collection

A single focus group was conducted at each of the five participating sites, and the same discussion guide was used to ensure that each session was conducted in a similar manner. The same experienced moderator facilitated all focus groups and was trained in the use of Colorectal Web as well as the computerized reminder system piloted in each site. Each focus group, lasting between 60 and 90 minutes, was audiorecorded and then transcribed verbatim by a professional transcriptionist. To protect the identity of focus group participants, the only demographic characteristics collected were role (clinician vs office staff) and gender.

### Analysis

To test for differences between participating (n=5) and nonparticipating (n=7) practice sites, two-tailed Student’s *t* tests were employed.

The focus group transcripts were analyzed using an iterative process to identify overarching patterns and salient themes. Using four main topics from the discussion guide as an initial organizing framework, the authors carefully read and analyzed the transcripts using the immersion/crystallization method [24]. This method requires those reviewing the transcripts to immerse themselves in the data by closely reading the transcript in great detail, followed by periods of reflection and deliberation to identify patterns and themes. Overarching patterns, themes, and subthemes were documented by the study’s investigators and then submitted to the principal investigator (PI). The PI then integrated these themes into a cohesive framework by identifying consistency between investigators and corroborating these themes with salient text. Next, each investigator reviewed this integrated summary, and discrepancies between the PI’s framework and investigators’ interpretations were negotiated until 100% agreement was achieved. The framework was then used to systematically review each transcript to identify inter-site variation. To illustrate recurrent themes, subthemes, and variation between sites, representative and especially salient quotations were selected (a complete summary of quotations is available upon request).

The following convention was used to indicate the frequency in which a particular theme or subtheme was expressed: a small number = one to two clinicians or one to two clinic staff, a moderate number = three to four clinicians or three to four clinic staff, a majority = five or more clinicians or five or more clinic staff. Direct quotes from respondents are presented in italics. To help minimize the length of quotations, ellipses are used liberally (three periods indicate a break within a sentence and four periods a break between sentences). Some longer quotations are included to ensure speakers’ meaning or context remains intact.

## Results

### Overview

Differences between the five participating and seven nonparticipating sites are presented in Table 1. All sites specialized in family medicine and were distributed throughout Michigan. With one exception—where participating sites were less likely to have self-pay patients compared to nonparticipating sites—the small sample size limited our ability to detect statistically significant differences. The participating sites tended to have fewer clinicians (physicians and nonphysician clinicians), higher clinician-to-patient ratios, larger proportion of patients from racial/ethnic minority groups, and more patients covered through managed care.

Demographic characteristics of participating sites and of focus group participants are outlined in Table 2. Two sites were located in suburban communities in the state’s west central or east central regions, one site was in a rural Upper Peninsula community, and two were in rural communities in the state’s east central or southern regions. As shown, one rural and one suburban site utilized EHRs. Each site was independently operated; that is, the sites were neither affiliated with one another nor with a larger health system. Among clinicians, five were male and four were female; all nonclinical office staff were female.

During focus group sessions, clinicians more actively shared their perspectives when compared to nonclinical staff—approximately 65% of comments came from clinicians whereas about 35% came from nonclinical staff. Moreover, while all clinicians were active participants in their respective focus group, more than half of nonclinical staff (n=10) remained silent throughout the entire focus group session. As shown in Table 2, nonparticipation was evenly distributed across sites and did not readily correlate with practice setting, use of EHRs, or clinicians’ gender. Salient themes and subthemes are outlined below according to the four main topics from the discussion guide.

**Table 1:** Comparison of the focus group sites and non-participating sites.

		Focus group		Nonparticipating	
		Practices (n=5)		Practices (n=7)	
Characteristics		Range	Mean	Range	Mean
**Providers**					
	Physicians	1-5	2.8	0-12	3.9
	Non-physician clinicians	0-2	1.0	1-2	1.3
Mean number of patients seen per day per practice		30-80	43.6	18-150	57.1
**Patient gender, %**					
	Female	50-60	57.0	55-65	59.2
**Patient race/ethnicity, %**					
	White	50-98	78.2	78-99	91.9
	Latino	0-20	6.4	0-7	1.5
	Black	0-20	4.3	0-6	1.4
	Other	0-10	2.7	0.8-14	5.3
**Patient insurance, %**					
	Medicare	14-48	28.4	1-40	22.8
	Medicaid	5-30	15.4	5-50	22.7
	Managed care	0-35	22.6	0-25	11.0
	Traditional indemnity	12-45	28.0	1-50	21.9
	Self-pay^a^	5-8	5.6	6-27	15.4

^a^Mean difference significant at the .05 level; *t*_10_=2.76, *P*=.02 (2-tailed independent samples *t* test, unequal variance).

**Table 2:** Characteristics of the participating practice settings, EHR use, and focus group participants’ professional status and gender.

Practice	Setting	EHR	Composition of participants
A	Suburban; west central Michigan	Yes	2 male physicians
			3 female staff (1 participated)
B	Rural; east central Michigan	Yes	1 female physician, 1 male physician
			5 female staff (2 participated)
C	Suburban; east central Michigan	No	1 male physician
			1 female physician assistant
			4 female staff (1 participated)
D	Rural; southern Michigan	No	1 female physician, 1 male physician
			3 female staff (2 participated)
E	Rural; Upper Peninsula Michigan	No	1 female physician
			3 female staff (2 participated)

### Current Colorectal Cancer Screening Approaches

#### General Approach to Colorectal Cancer Screening

A majority of clinicians reported discussing CRC screening with patients during preventive visits; such visits were described as a natural forum for sharing health information and soliciting patient input. A majority of clinicians reported that they had their own particular methods for remembering to address CRC screening, such as a notation or reminder on the patient’s chart or a more general system based on patient age. However, a moderate number reported not always remembering to bring CRC screening up during the clinical encounter. For example, the clinician in Site E explained: “…it’s somewhat hit or miss.” All clinicians in the two sites with EHRs (Sites A and B) reported their system provided a computerized reminder that enabled them to be more aware of patients’ preventive needs. A clinician in Site A commented: “We have a lot of little chart reminders. Things that are in the chart remind us, oh this person needs a colonoscopy.” In the remaining sites, clinicians described having to review the previous progress notes and/or flow sheets to determine if patients were up to date on preventive services. Clinicians and staff from Site C developed a reminder system that triggered clinicians through the use of a visual cue: they stapled FOBT kits to posters and placed them in examination rooms. Despite this, one clinician noted: “It’s kind of hard to keep track of everything….[For [s]ome people it just didn’t work. It doesn’t matter what you did.” Only one site (Site A) reported using a list provided by health insurance companies to identify patients who are due for CRC screening. At this site, office staff contacted the identified patients using letters and telephone calls to inform the patient that it was time for a screening.

#### Shared Decision Making and Colorectal Cancer Screening

A majority of clinicians described that the primary resource used to facilitate CRC screening discussions was their own interactions with patients during the clinical encounter; a moderate number expressed a need for additional educational tools that could help facilitate more effective discussions, such as brochures and pamphlets (Sites C and D) and video vignettes (Site E).

A moderate number of clinicians reported that when discussing CRC screening with patients, a good portion of the conversation tended to focus on communicating the relative advantages and disadvantages of each screening method. A majority of clinicians, however, revealed they commonly recommended the patient undergo the CRC screening method that the clinicians themselves preferred rather than presenting all options. For example, clinicians at sites A and D primarily recommend colonoscopies, whereas clinicians at sites B and C almost exclusively recommended FOBTs. As indicated by one clinician from site A, “The culture has evolved over the last number of years where colonoscopies was [sic] probably the standard of care.” As reported by a majority of clinicians and staff, the principal barriers to patients’ access to CRC screening were lack of insurance and concern about costs. A majority of clinicians also described time constraints as a significant barrier. As reported by the clinician from site C, “I don’t really want to discuss it. I just want to do it….I don’t feel in my office [that] I [could] sit down and have a five or ten minute discussion of the pros and cons of hemoccults vs colonoscopies.”

### Perceptions/Opinions About Colorectal Web

#### Overall Impression

Clinicians and staff at all sites agreed that Colorectal Web was an informative and educational tool they could easily recommend to their patients; moreover, clinicians from Sites C, D, and E affirmed they would start using it immediately if it were available to patients through the Internet. Clinicians from Site A, however, indicated that a decision aid—Web-based or otherwise—was superfluous because they subscribed to a general belief that colonoscopies are the most appropriate standard for care. While clinicians from Site B described being impressed with the tool, they reported a concern that many of their patients did not have Internet access or the requisite technical skill to complete a Web-based tool.

#### Facilitators

A majority of clinicians and a moderate number of staff asserted that Colorectal Web is an efficient source of CRC screening information. Because of this, the website was described as having particular utility for those patients who have many questions and want to sift through a lot of information. A small number of clinicians also emphasized that Colorectal Web could be used as a resource for patients to learn more about a procedure even after a preferred screening modality had been identified. This reaction was endorsed even by clinicians from Site A, who believed the Web-based tool could help educate patients about colonoscopies and ease their discomfort about the procedure. In describing communication style with patients about the topic of colonoscopies, one clinician from Site A asserted: “It’s usually more a persuasion. Telling them what it’s really like and it’s not as bad as you think probably and explaining the test and what they have to think about and try to convince them to do it.” A moderate number of clinicians described that Colorectal Web could be an especially useful tool for patients concerned about the out-of-pocket expense of screening because it helps users weigh the pros and cons of each screening option through the lens of its monetary cost. A majority of clinicians felt Colorectal Web could help to improve clinical efficiency, as patients’ questions about CRC screening could be answered through using the tool prior to the office visit. A typical response was expressed by the clinician in Site E: “Big time saver, I think.”

Although a purported benefit of a decision aid is improved shared decision making, none of the clinicians mentioned this as a potential benefit; rather, the decision aid was seen as a way to cut back on the need to discuss CRC screening with the patient. A notable quote was made by the clinician in Site C: “Well, we don’t really want to…can I be frank and open? I don’t really want to discuss it [ie, screening options]. I just want to order it. Just like I say CBC. This is time to do this.”

#### Barriers

A recurring theme from a majority of clinicians and staff was a concern that many patients may not have regular access to the Internet and therefore would not be able to utilize the Web-based decision aid. The proportion of patients described as having access to the Internet varied by site, with the lowest reported rate being about 5% and the highest about 60%. Moreover, a majority of clinicians argued that relatively few patients were regular users of the Internet, noting that Web-based tools may not necessarily be the preferred method for making health care choices among those aged ≥50 years. Relatedly, a majority of clinicians and staff underscored that some older patients do not have the requisite skillset to use a Web-based tool. For example, a clinician in Site B noted:

[many patients have] no comfort with computers, and I can’t see more than perhaps 15, 20% of our total patient population even being able to access that. And of that, [we would be lucky] if we had 5 or 7% that would actually do it to the level that you are discussing....[F]or a certain group of patients that would be a wonderful tool. I don’t think it would be for our group of patients primarily because we have a large percentage of low functioning patients.

The majority of clinicians did not have a clear idea of what percentage of their patients were Internet users, and none had actually assessed their patients’ Internet usage formally. The general consensus among clinicians was a belief that their older patients (those over age 65) were particularly low utilizers of the Internet. Patients’ Internet literacy or interest in using the Internet was also a concern, particularly in regard to the utility of Colorectal Web as an educational tool. As summarized by a clinician in Site C: “You need a killer application. You need to have people [to] have a reason they want to get on the Web. They have to want it before you can get them to use it.”

### Feasibility of Implementing and Linking Colorectal Web to a Computerized Reminder System

A moderate number of clinicians and staff endorsed the belief that integrating the Web-based decision aid with a computerized reminder system would be an efficient way to manage patients’ CRC screening preferences. In addition, they perceived integration to be a natural progression of existing health information technology. However, about an equal number reported concerns that integration might not occur seamlessly given their existing technology and daily workflow. Expressing both excitement about the technology and concern about its integration, a clinician from Site E opined: “From my perspective as a physician I really enjoyed [the clinician reminder system] because it really reminded me at all visits to discuss with people…if they have had it or not had screening. So from my aspect it was awesome, but not everybody shares the same opinion about it as I do because we had a lot of computer issues with it.” A clinician from Site A described doubt about the benefit of electronic decision aids, citing a belief that their use was being driven primarily from external pressures rather than clinical utility: “[T]o be honest, I don’t know if these tools would be a benefit to us anymore. Because again, it’s just a high priority on the insurance companies…I don’t know how useful something like this would be.”

### Anticipated Influences on Practice Workflow and Logistics

Clinicians and staff from each site reported a preference for patients to use Colorectal Web prior to their scheduled office visit. A majority of them believed that it was neither practical nor efficient to have a computer in the waiting area for patients to complete the tool. In addition to expressed concerns about patient confidentiality, concerns about an onsite computer included worry that children might use the computer or that patients would require assistance and thereby distract staff from other duties and responsibilities. One clinician from Site B expressed concern about patients damaging the computer or trying to steal it: “It would be destroyed. Correct. It would be destroyed or it would be stolen....[like] the flowers and all the pictures on the wall.” A staff member from Site C was concerned that computers with Web access might be used for inappropriate purposes, thereby requiring constant supervision from staff: “Yeah, it would definitely have to be limited on…access and everything. We wouldn’t want people accessing porn sites or anything out in the waiting room.”

There was also an expressed concern that patients’ use of the tool while in the waiting area may interrupt the clinics’ workflow; for example, if patients arrived late or if the clinic was running ahead then patients could be stuck using the tool when it came time to meet with the clinician. One possible solution to this timing problem, as suggested by a small number of participants, would be for patients to access the tool on a tablet computer that could be carried around by the patients throughout the clinic. As an alternative to using Colorectal Web within the clinic, a majority of clinicians and staff endorsed the idea of using mail-based postcard reminders to prompt patients’ use of the tool at home and in advance of their schedule appointment. A moderate number of clinicians and staff described already having a system in place for sending patients reminders for appointments for physicals; however, there was no standard for the timing of reminders or for sending reminders that specifically addressed CRC screening.

With the exception of Site A, where no comments on the topic were made, clinicians from each site believed that written materials (eg, information sheets, brochures, informational cards) would be beneficial to help educate patients about CRC and the range of screening options. In fact, a moderate number of focus group participants expressed a desire to have more written materials that could help facilitate discussions about CRC screening, both to save time during the visit and to improve patient’s knowledge. While Colorectal Web was lauded for its interactive format and its ability to help patients weigh their own values against the various screening options, written materials were generally regarded as the gold standard for conveying information to patients outside of the context of the clinical encounter. A tri-fold brochure that included a table comparing and contrasting the range of CRC screening options was endorsed by a majority of clinicians and staff as having utility both in and outside of the clinical setting. Brochures were also cited by a small number of clinicians and staff as having benefit due to their perceived cost and ease of use—as stated by one clinician from Site D: “[Pamphlets are] more cost effective and easier, plus the pamphlet...they can still look at when they go home.”

When asked about a paper/pencil alternative to Colorectal Web (eg, a CRC preference workbook), both clinicians and staff rejected it as overly cumbersome. They reported a belief that patients would not like to fill out additional paperwork as there are already many forms being completed by patients inside and outside of their appointments. The one exception was for patients who live in rural communities where high-speed Internet access may be limited. For these patients, a workbook-style corollary to the Web-based tool could be given to patients for completion before their next appointment. A small number of clinicians suggested that patients using the workbook should be encouraged to discuss it with their clinician either at the next appointment or through another agreed upon method (eg, phone call).

## Discussion

### Principal Findings

Our study leveraged a unique opportunity to enroll clinicians and staff from a diverse set of community settings to ascertain how a Web-based and interactive decision aid might impact CRC screening practices and the clinics’ daily workflow. With few exceptions, focus group participants generally agreed that promoting patients’ use of the decision aid *prior* to the clinical visit would be an effective way to educate and activate patients so they can make informed screening decisions in light of their most salient concerns. While the majority of clinicians endorsed Colorectal Web as a promising tool, enthusiasm was neither universal nor without restrictions. Two commonly cited concerns were patients’ limited computer literacy and lack of access to the Internet. This worry was especially salient for those serving a predominantly low-income population. In addition to Internet access, some focus group participants noted concern that the tool might not be sufficiently interesting to motivate patients to use it. While access and interest could be countered, in part, by directing patients to the tool while waiting for their appointment in the clinic, making the tool available to patients during their office visit was largely perceived as unwieldy and time-prohibitive. One of the more salient concerns—especially from office staff—was that patients’ use of the Internet would need to be closely monitored (eg, restricting access to inappropriate sites). From this perspective, onsite access to the Web-based tool was perceived as increasing the clinics’ workload rather than facilitating shared decision making and optimizing CRC screening. Though there are a number of tools designed to limit users’ access to certain Internet content, their use as a possible solution was not raised during the focus group sessions.

While findings from previous research suggest patients’ access to high-quality decision aids is accelerating, decision aids continue to be underutilized in community-based primary care settings [6,7]. Commonly cited barriers to decision aid use include time constraints, lack of fit between the aid and patient need, and a poor match between the aid and the demands of the clinical setting [6,7,25,26]. Findings from our study add to the literature by providing a nuanced view of perceived barriers and facilitators, and how an interactive Web-based tool could be integrated into community-based settings. Results show that not all focus group participants believed an electronic, interactive decision aid linked to a computerized reminder system would have enough advantages over traditional paper resources to justify their use in the clinical setting. Interestingly, the sites utilizing EHRs were just as likely to have expressed these concerns as those not using such technology. While potentially reducing concerns related to timing and mobility (eg, patient stuck at a desktop completing the decision aid when it was time for them to see the clinician in the exam room), there was disagreement among focus group participants as to whether tablet devices would be an effective solution. Tablets were described to have their own set of limitations, including cost, maintenance, patients’ literacy with the technology, and keeping track of the devices while patients are coming and going throughout the day.

Despite a growing literature suggesting Web-based decision aids may be superior to other decision aid modalities in improving patient knowledge and behavioral outcomes [27-32], the focus group participants in this research were concerned their use could negatively impact their site’s day-to-day workflow. This finding complements Schroy et al, who found a majority of surveyed primary care providers were either neutral to or disagreed with the statement that a Web-based decision aid would be easy to implement in their practice [33]. Findings from focus groups also correspond to conclusions reached by Légaré et al, who after reviewing the literature on strategies to improve the use of decision aids by health care professionals could not draw firm recommendations for the most effective dissemination strategy [26]. Rather than a continued focus on the creation of new decision aids per se, future research should focus on improving our understanding of how existing decision aids can be integrated into daily practice.

As suggested by others, current policies that shape the nation’s health care system will likely need to change before clinicians fully embrace shared decision making and the tools (eg, interactive decision aids, computerized reminder systems) that promote it [6,34-36]. Several candidate policies to be changed include redefining medical necessity so that it better includes the principle of an informed patient; creating economic incentives (and reducing disincentives) for shared decision making; establishing a clear legal standard that facilitates shared decision making and informed patient choice; developing effective systems-based processes that promote decision aid uptake and utilization; and modifying health care accreditation standards to account for the use of tools that promote a patients’ ability to make educated, values-based decisions about care when more than one medically reasonable treatment option exists [7,35-40].

Consistent with previous research, a moderate number of clinicians in our study preferred one CRC screening modality over all others [7,38-41]. This finding is troubling given evidence that providing patients with options increases screening follow-through, while a more autocratic recommendation—where one screening modality is strongly favored over another—may act to limit screening follow-through [6,42]. Future studies should seek to elucidate the relationship between clinicians’ practice style and patients’ screening follow-through by examining patient-clinician encounters directly and assessing how decision aids affect communication, treatment decisions, follow-through, and health outcomes.

Focus group participants from the five community practice sites in this study reported challenges similar to previously published findings about providing opportunistic preventive services: lack of time due to acute or chronic care needs, administrative obstacles, patients’ psychosocial limitations (eg, literacy), and clinicians’ treatment preferences [42-46]. Findings revealed that clinicians at each site were prone to perceive the provision of opportunistic care as daunting rather than an opening to improve the overall quality of care. The clinicians in sites utilizing EHRs were more open to providing opportunistic preventive care, and although this must be interpreted with caution due to the small sample size, it may suggest that the successful integration of electronic health information technology such as computerized reminder systems may mediate clinician behavior when the flow of information is relatively seamless. For those sites not actively using EHRs, implementing a new layer of health information technology to communicate patients’ screening preferences must be carefully integrated into the sites’ existing clinical processes so as to minimize disruptions to productivity and workflow.

### Limitations

There are a number of limitations to this study. First, each site was affiliated with the Great Lakes Research Into Practice Network, and all sites had previously participated in a study investigating the implementation of a computerized reminder system. Clinicians and staff at these sites may therefore not represent those less engaged in health services research or those more opposed to the adoption of electronic health information technology. This said, by being early adopters of technology, our sample was likely primed to describe barriers and facilitators to the technology’s implementation rather than to the use of Web-based technology itself. Second, the study focused on a limited number of small community practice settings; therefore, findings may not be generalizable to larger practice settings or settings that have a close affiliation with a specific health system. Third, our sample of five sites, 9 clinicians, and 18 staff (of which only 8 staff actively participated) was relatively small. Because the intent of this qualitative study was to solicit rich descriptions informed by the give-and-take dynamic of the focus group, increasing the number of sites was both impractical and cost prohibitive given the study’s limited resources. Importantly, because no novel findings were identified by the fifth focus group, thematic saturation was likely achieved. Informed by findings from this research, follow-up studies might consider employing a survey-based methodology to reach a larger and nationally representative sample. Fourth, because of the small sample and to ensure respondents’ confidentiality, we did not attempt a more nuanced stratification of responses based on participant demographics. It may be that a persons’ background or demographic profile primes or limits ones’ readiness to accept new technology or change clinical behavior; for example, it is possible that older physicians, like older patients, may be less comfortable with using new technology when compared to their younger counterparts. Investigating the possible role of background or demographics on ones’ readiness/willingness to use new forms of health information technology should be the focus of future research. Fifth, over half of the nonclinical staff failed to actively participate in the focus groups. It may be that some staff were uncomfortable sharing their thoughts in front of clinicians, who in some cases were the staff members’ employer. It is also possible that some staff (eg, an office biller) may have been so far removed from the day-to-day clinical milieu that they simply had nothing substantive to add. Focus groups separating clinicians and staff may yield different findings and could be explored in future research. Sixth, the issue of tailoring screening according to the patient’s CRC risk (eg, recommending colonoscopy only to those with increased risk vs offering options to those with average risk) was not discussed. Incorporating patients’ CRC risk in CRC screening discussion is important, and our current website (now renamed as ColoDATES and tested in the field in a federally funded study), includes an interactive risk assessment tool [47]. Seventh, the focus groups were conducted in 2006 and 2007 and therefore do not necessarily reflect the increased use of EHRs observed over the past few years. Findings from the 2011 National Ambulatory Medical Care Survey reveal, however, that nearly 40% of United States primary care physicians practice in sites without an EHR, and only 22% practice in sites with fully functional systems [48]. Moreover, the use of Web-based interactive decisions aids linked to a computerized reminder system remains on the cutting edge of electronic health information technology. And last, the five focus group sessions documented clinician and staff member perceptions and not their actual behavior within the clinical setting. It is possible that participants may have under- or overestimated barriers and facilitators based on their personal biases toward electronic health information technology. Likewise, because patients themselves were not included in this research, it is possible that participants may have under- or overestimated patients’ barriers to accessing or utilizing Web-based tools. Given previous findings that clinicians may sometimes overestimate their own performance [49,50] and underestimate patients’ literacy [51-53] and financial status [54-56], findings should be interpreted with caution.

### Conclusions

The clinician and staff participants in this research perceived the interactive and Web-based decision aid as a promising tool for informing patients about the range of CRC screening options. One benefit ascribed to the tool was that it could be utilized by patients outside of the face-to-face clinical encounter. After reviewing the online decision aid, participants agreed that patients would be well informed about the pros and cons of each CRC screening modality, which could help to increase the sophistication of dialogue between patients and clinicians. Moreover, linking the tool to a computerized reminder system was described as a potentially effective way to inform clinicians of patient’s screening preference, and the reminder could be used to trigger conversation and promote shared decision making. However, focus group participants also voiced concerns regarding patients’ computer and Internet literacy and disruptions to the clinics daily workflow. These concerns—as well as solutions to overcome them—should be the focus of future research.

Web-based CRC screening decision aids and linked computerized reminder systems hold promise for improving patient-clinician communication and subsequent follow-through with screening, but only if the linking fits seamlessly into the clinical setting. Our findings suggest the trend toward adopting electronic health information technology—including the growing mandate to implement and achieve meaningful use of EHRs—must not only focus on improvements to the technology itself, but also integration of the system-related processes that enable the technologies’ successful adoption. Close attention to systems and processes has particular importance in small community-based practice settings where resources in time, staff, and money are likely to be limited.

## Abbreviations

CRC: colorectal cancer
EHR: electronic health record
FOBT: fecal occult blood test
PI: principal investigator
